# Assessing the Usability and Effectiveness of an AI-Powered Telehealth Platform: Mixed Methods Study on the Perspectives of Patients and Providers

**DOI:** 10.2196/62742

**Published:** 2024-11-25

**Authors:** Ekta Jain, Srishti Gupta, Vandana Yadav, Stan Kachnowski

**Affiliations:** 1 Healthcare Innovation and Technology Lab New York, NY United States; 2 Indian Institute of Technology Delhi Delhi India

**Keywords:** usability study, telemedicine, web platform, patient-provider feedback, artificial intelligence, AI triage

## Abstract

**Background:**

Telemedicine has revolutionized health care by significantly enhancing accessibility and convenience, yet barriers remain, such as providers’ challenges with technology use. With advancements in telemedicine technologies, understanding the viewpoints of patients and providers is crucial for an effective and acceptable telemedicine service. This study reports the findings on the usability and effectiveness of the HelixVM artificial intelligence powered platform, analyzing key aspetcs like asynchronous health care, access, time efficiency, productivity, data exchange, security, privacy, and quality of care from patient and provider perspectives.

**Objective:**

This study aims to assess the usability and effectiveness of the HelixVM marketplace platform.

**Methods:**

We recruited 102 patients and 12 providers in a mixed methods study design involving surveys and in-depth structured interviews with a subset of the providers. The survey questionnaires are a modified version of the Telehealth Usability Questionnaire. We analyzed patient data using descriptive statistics and exploratory factor analysis to identify latent demographic patterns. For provider data, we used a deductive thematic analysis approach to identify key themes from the interviews and interpreted overall sentiments of the providers as negative, neutral, or positive. We also calculated percentages of different provider responses from the survey and interviews, where applicable.

**Results:**

Overall, 86.3% (88/102) of the patients reported satisfaction with HelixVM, and 89.2% (91/102) indicated that they would use the services again. A total of 91.1% (93/102) of the patients agreed that HelixVM improves access to health care and is an acceptable way to receive health care, and 98% (100/102) agreed it saves time. Chi-square tests demonstrated statistical significance for all survey questions (*P*<.001). The results from factor analysis show a higher propensity of middle-aged women, who had a fast-track encounter type, who self-reported a medium level of technology savviness, and who are residing in the South region of the United States rating the platform more positively. With regard to the providers, the thematic analysis identified themes of asynchronous medicine in terms of the accessibility and quality of care, time and productivity, integration within the workflow, data exchange, and artificial intelligence triage. Certain challenges regarding incomplete data in patient charts and its impact on provider time were cited. Suggestions for improvements included options to ensure the completeness of patient charts and better screening to ensure that only asynchronous, *qualified* patients are able to reach the provider.

**Conclusions:**

Overall, our study findings indicate a positive experience for patients and providers. The use of fast-track prescription was considered favorable compared to traditional telemedicine. Some concerns on data completeness, gaps, and accuracy exist. Suggestions are provided for improvement. This study adds to the knowledge base of existing literature and provides a detailed analysis of the real-world implementation of a telemedicine market-place platform.

## Introduction

### Background

Access to health care is a fundamental necessity; yet, for many individuals, it remains an elusive privilege. Factors such as financial constraints, time limitations, and geographical distance often serve as insurmountable barriers, rendering health care services inaccessible to those who need them most. Telemedicine emerges as a solution to this problem, using information and communication technologies to remotely diagnose, treat, and care for patients. It has revolutionized health care by significantly enhancing accessibility [1]. While telemedicine has existed for decades, its uptake has been hindered by limitations in telecommunication infrastructure, digital literacy, and access to the necessary devices. However, the COVID-19 pandemic accelerated the global adoption of telemedicine, establishing it as a common health care delivery practice even beyond the pandemic’s peak. Both patients and providers now recognize telemedicine’s benefits in delivering health care, saving time and money, and enabling the swift treatment of conditions [2].

Numerous studies have explored telemedicine from both patient and provider perspectives, examining its advantages, capabilities, challenges, and barriers across various medical specialties [3,4]; for instance, recent studies have focused on understanding how telemedicine shaped health care access and delivery during the COVID-19 pandemic where practitioners reported varied experiences with telemedicine, concerns over conflicting guidance, and increased workload. Patients appreciated the convenience and reduced infection risk but expressed concerns about costs. Both patients and providers agreed that telemedicine can reduce unnecessary in-person visits [5]. Collaboration among practitioners increased, allowing the easy sharing of medical records [6], and practices adapted to remote services [1]. Certain barriers faced by providers have been identified. These include concerns regarding privacy and confidentiality, cases of misdiagnosis, misuse of the system to ignore notes and other assistive information, and ensuring the accuracy of data [6,7]. Importantly, technical difficulties pertaining to the software are a prevalent barrier among health care providers [5].

With advancements in information and communication technologies, understanding how innovations such as artificial intelligence (AI) and machine learning are reshaping telemedicine is crucial. AI has the potential to significantly improve health care professionals’ capabilities and enhance telemedicine’s efficiency and safety; for instance, by leveraging AI algorithms, telemedicine platforms can address avoidable medical errors, streamline clinical workflow processes, and provide more precise and personalized diagnoses. In addition, AI enables real-time analysis of patient data, aiding in scientific decision-making and optimizing resource allocation. It also facilitates remote patient monitoring by detecting abnormalities and changes from various data sources, such as wearable devices. With personalized treatment recommendations, AI enhances patient care, improves outcomes, and promises to increase health care quality, reduce costs, and ensure equitable access, regardless of location or mobility constraints [8].

Many research endeavors have explored the viewpoints of both health care providers and patients regarding telemedicine, particularly with the evolution of technologies such as AI and machine learning. Understanding these viewpoints is crucial in the evolving landscape of telemedicine. This study reports findings from a usability and effectiveness study of HelixVM, a telemedicine platform that uses an AI-powered triage system for health care delivery. Essentially, it is a web-based health care marketplace that addresses various health care needs, offers fast-track prescriptions, and provides medical opinions accessible from anywhere, irrespective of insurance status. The platform enhances provider capacity without additional staff, ensuring smooth interoperability with electronic medical records (EMRs) for effective patient management and secure data handling. HelixVM delivers both FastTrack Rx asynchronous medicine and traditional synchronous video telehealth. FastTrack Rx simplifies the prescription process by allowing users to select their payment method, provide personal details and medical history, complete an AI-guided symptom questionnaire, have their information reviewed by a medical team, and receive their prescription promptly at their preferred pharmacy for swift access to care.

### Study Approach

To assess HelixVM’s real-world utility and effectiveness, we conducted a sequential exploratory mixed methods study surveying 102 patients and 12 providers. In addition, we collected data on the effectiveness of the platform from 10 (83%) of the 12 providers using structured 1-on-1, in-depth interviews. This comprehensive approach provided deep insights into the usability, user experience and effectiveness of the platform, and on the various aspects of asynchronous medicine. We discuss these findings, particularly asynchronous medicine, health care accessibility, saving time, productivity, data exchange, security, privacy, AI triage, and quality of care.

## Methods

### Overview

This mixed methods study consisted of 2 phases: a quantitative survey administered to patients and health care providers and structured, in-depth, 1-on-1 interviews with a subset of the providers to gain further insight into the effectiveness of the platform. A total of 102 patients and 12 health care providers participated in the study’s survey phase. Subsequently, 10 (83%) of the 12 providers who completed the survey were recruited for structured in-depth, 1-on-1 interviews.

### Participant Recruitment

The sampling frames for patients and providers were provided by HelixVM, and participants were recruited electronically via email. Initially, the patient sampling frame consisted of 109 individuals. To improve recruitment rates and save time, HelixVM provided additional sampling frames—one with 122 patients and the other with 924 patients—at 2 different time points. When the number of individuals who responded and consented reached 105, we ceased further enrollment. Of the 1155 patients, 102 (8.8%) were successfully recruited into the study. Of the 16 providers in the sampling frame, 12 (69%) were recruited for the survey. Of these 12 providers, 10 (83%) were recruited for the interviews on a first-to-respond and complete participation basis.

### Data Collection

#### Phase 1

A modified version of the Telemedicine Usability Questionnaire (TUQ) [9] was used to assess the usability of the HelixVM platform among both patients and providers. The questionnaire coveres aspects of convenience, quality of health care, ease of use of the system, error resolution, and satisfaction. All statements in the questionnaire were rated on a 7-point Likert scale (1=*strongly disagree*, 2=*disagree*, 3=*somewhat disagree*, 4=*neither agree nor disagree*, 5=*somewhat agree*, 6=*agree*, and 7=*strongly agree*). User technology savviness and the likelihood of recommending the platform to others were measured on an 11-point scale ranging from 0 to 10. The detailed questionnaires are available in Multimedia Appendix 1. The Cronbach α values of the subscales in the original TUQ have been reported previously [9]. We calculated the Cronbach α values of the modified TUQ subscales used in this study using the *ltm* package in R (R Foundation for Statistical Computing). The questionnaires were administered electronically using Microsoft Forms and responses stored in real time.

#### Phase 2

Approximately 30 days after the surveys were completed, 10 (83%) of the 12 providers who participated in the surveys were recruited for in-depth, 1-on-1 structured interviews. The interviews consisted of 15 questions to collect data on HelixVM’s value propositions such as the AI triage system, integration with the EMR, data exchange and safety, and improvements to the platform, as well as the overarching themes of asynchronous medicine, provider time management, and compensation. The provider interview questions guide is available in Multimedia Appendix 2.

### Eligibility Criteria

The eligibility criteria are presented in Textbox 1.

**Eligibility criteria.**

**Patients**

- Individuals who are registered users of the marketplace platform developed by HelixVM
- Individuals who have been active users (used the platform at least once in the last 1 year)
- Individuals aged >18 years
- Individuals who are able to understand study background and provide informed consent

**Providers**

- Any provider who is an authorized provider on HelixVM’s marketplace platform
- Providers who have been active users in the last 1 year
- Providers who have used the platform for ≥2 months

### Data Analysis

#### Overview

For the quantitative data, we performed descriptive and statistical analyses (factor analysis), while the qualitative data were analyzed using thematic and sentiment analysis as detailed in the following subsections.

#### Patient and Provider Descriptive and Statistical Analyses (Phase 1)

For all survey responses, we calculated overall survey response percentages as well as percentages stratified by sex, age group, and encounter type. We also calculated a net promoter score [10] using the scores on the respondents’ likelihood of recommending the HelixVM platform to others. For the patient survey responses, we performed exploratory factor analysis [11] to identify latent patterns. We coded the patient and provider survey responses on an ordinal scale treating the neutral category as the midpoint as follows: 1=*strongly disagree*, 2=*disagree*, 3=*somewhat disagree*, 4=*neither agree nor disagree*, 5=*somewhat agree*, 6=*agree*, and 7=*strongly agree*. For factor analysis, as the data were ordinal categorical, we applied polychoric correlations and used the minimum residual estimation method for the factor loadings. To obtain rotated factor loadings, we applied varimax rotation. Age was recoded into the following groups: >18 to <30, ≥30 to <45, and ≥45 to 65 years. Technology savviness scores were recoded into the following categories: low (0-4), medium (5-7), and high (8-10). In addition, US states were grouped into Northeast, South, Midwest, and West regions based on the US Census Bureau’s regional classifications [12]. To ensure the reliability and credibility of the factor analysis, we calculated the Kaiser-Meyer-Olkin statistic. For the patient questionnaires, we assessed internal consistency using Cronbach α value for the modified TUQ. For the patient data, we calculated *P* values at the 5% significance level to test differences in response percentages both overall and when stratified by sex, age group, technology savviness, encounter type, and state. For the provider dataset, due to the small sample size, we did not perform *P* value calculations.

#### Provider Interview Analysis (Phase 2)

We analyzed provider interview data using deductive thematic analysis [13,14] to identify and interpret meanings or themes. We also performed sentiment analysis to assess the providers’ emotional disposition toward the HelixVM platform. For *yes* or *no* questions, we conducted a quantitative analysis by calculating the percentage distribution of responses for each category.

For qualitative analysis, we used data triangulation, which is a strategy to test validity through the convergence of information from different sources and enhance the reliability of the findings [15]. Data triangulation was used to enhance the validity and reliability of our findings by comparing responses from the structured interviews with those from the survey. The survey, which included basic usability questions, provided quantitative data that reflected provider experiences with the HelixVM platform on key usability aspects such as ease of use, time savings, and overall satisfaction with the platform. The provider interviews, by contrast, offered in-depth qualitative insights into these same usability aspects. During the analysis, we systematically compared the survey results with the interview responses to identify patterns, themes, and any discrepancies; for instance, when a provider strongly agreed in the survey that “HelixVM improves access to health care services,” we cross-referenced this with their detailed interview responses to see whether similar sentiments were expressed or whether any additional context was provided. In this study, data were collected from providers of different backgrounds for multiple perspectives. In addition, by incorporating multiple data sources such as surveys and interviews, we aim to deepen our understanding of the usability, user experiences, and effectiveness of the HelixVM telemedicine platform. Final themes from the qualitative provider interviews were triangulated with survey data for a nuanced understanding and interpretation of the findings. This process involved iteratively categorizing similar survey and interview questions and responses to identify common themes and patterns. All statistical and quantitative analyses were performed in R (R Foundation for Statistical Computing).

### Ethical Considerations

This study was approved by the BRANY (Biomedical Research Alliance of New York) Institutional Review Board (23-12-625-681). No personally identifiable data were used in the analysis. All data were stored on secure, Health Insurance Portability and Accountability Act (HIPAA)–compliant servers at Healthcare Innovation and Technology Lab (HITLAB), accessible only to the core research team. Written informed consent was obtained from all participants using DocuSign before they were administered the survey questionnaire and before the 1-on-1 interviews.

The patients and providers were compensated with US $50 and US $100, respectively, in the form of eVISA gift cards for taking part in the surveys. The providers were compensated with an additional US $100 eVISA gift card for participating in the interviews.

## Results

### Descriptive Analyses

#### Patients

A total of 102 patients successfully completed the survey remotely using a web-based link. Their demographic details are presented in Table 1. As age was not a normally distributed variable, the median age and IQR are reported.

**Figure 1:**
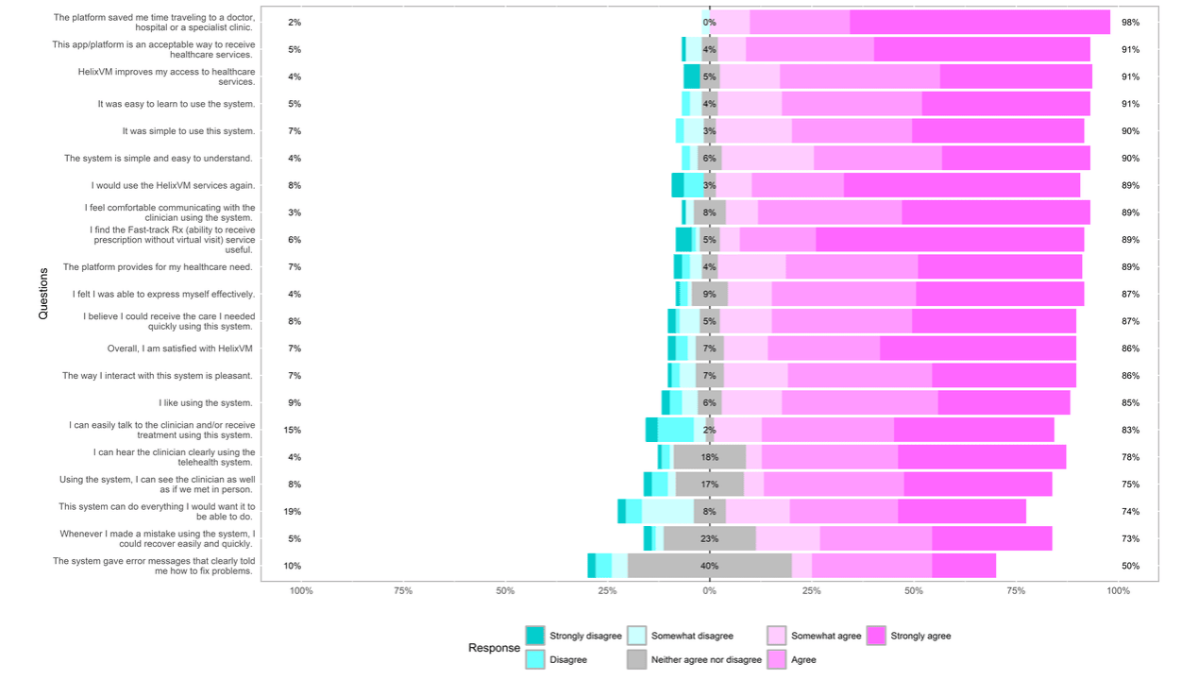
Percentage of responses from the patient survey questionnaire.

The Cronbach α values of the subscales of the modified TUQ were >0.7, indicating good reliability (Multimedia Appendix 3). The majority of the participants were female (72/102, 70.6%), aged 30 to <45 years (54/102, 52.9%), and had a fast-track encounter type (70/102, 68.6%). The median age of the participants was 37.6 years (IQR:12.75), and they were predominantly from the South region of the United States (69/102, 67.6%). The patients’ survey responses were calculated as percentages and are presented in Figure 1. Overall, 86.3% (88/102) of the patients reported satisfaction with HelixVM, and 89.2% (91/102) indicated that they would use the services again. Approximately 91.1% (93/102) and 98% (100/102) of the patients agreed that HelixVM improves access to health care and saves time, respectively. Approximately 91.1% (93/102) of the patients found the platform to be an acceptable way to receive health care and believed that it provides for their health care needs, while approximately 89.2% (91/102) found the fast-track prescription service useful.

In terms of simplicity of use, approximately 90.2% (92/102) of the patients agreed that the system was simple to use and easy to learn, as well as simple and easy to understand (Figure 1). Approximately 85.3% (87/102) of the patients liked using the system and found their interaction with the system pleasant. Regarding their interaction with the clinician during a virtual visit, approximately 81.4% (83/102) of the patients agreed that they could easily communicate with the clinician and receive treatment approximately 87.2% (89/102) were able to express themselves effectively, while 89.2% (91/102) said that they felt comfortable communicating with the clinician using the system. Approximately 81.4% (83/102) of the patients agreed that they could hear the clinician clearly, while approximately 76.5% (78/102) felt that they could see the clinician as well as they would in an in-person meeting. Overall, 73.5% (75/102) of the patients felt that the system could do everything that they would want it to be able to do. In terms of error recovery, 50% (51/102) of the patients felt that the system gave error messages (in cases such as a technical limitation or missing or invalid user information) that clearly told them how to fix the problems, and 76.5% (78/102) of the patients felt that the system allowed them to easily and quickly recover from mistakes made while using it.

Results from the goodness-of-fit chi-square tests demonstrated that the response percentages for all questions were significant at the 5% significance level (*P*<.001; Multimedia Appendix 4). For some of the survey questions, differences were observed between the male and female participants (Table 2), encounter type (Table 3), and state (Table 4). There were no statistically significant differences in responses for age groups and levels of technology savviness, that is, the *P* values for differences in responses across all statements in these demographic groups met the threshold for significance.

**Table 1:** Patient demographics (n=102).

Characteristic		Value
Age (y), median (IQR)		36.00 (12.75)
**Sex, n (%)**		
	Male	30 (29.4)
	Female	72 (70.6)
**Age group (y), n (%)**		
	>18 to <30	25 (24.5)
	≥30 to <45	54 (52.9)
	≥45 to 65	23 (22.6)
**Encounter type, n (%)**		
	Virtual visit	32 (31.4)
	Fast track	70 (68.6)
**Technology savviness, n (%)**		
	Low	37 (36.3)
	Medium	16 (15.7)
	High	49 (48)
**State (region), n (%)**		
	Northeast	12 (11.8)
	South	69 (67.6)
	Midwest	11 (10.8)
	West	10 (9.8)

**Table 2:** Sex-based differences for patient survey questions with significant *P* values (n=102).

Statements in the questionnaire			Sex, n (%)^a^					*P* value
			Male (n=30)		Female (n=72)			
“**The platform provides for my healthcare need”**								.03
	Strongly agree	9 (30)		32 (44)				
	Agree	13 (43)		19 (26)				
	Somewhat agree	2 (7)		16 (22)				
	Neither agree nor disagree	2 (7)		2 (3)				
	Somewhat disagree	2 (7)		1 (1)				
	Disagree	0 (0)		2 (3)				
	Strongly disagree	2 (7)		0 (0)				
“**It was easy to learn to use the system”**								.01
	Strongly agree	7 (23)		35 (49)				
	Agree	13 (43)		22 (31)				
	Somewhat agree	5 (17)		11 (15)				
	Neither agree nor disagree	3 (10)		1 (1)				
	Somewhat disagree	0 (0)		3 (4)				
	Disagree	2 (7)		0 (0)				
“**Simple and easy to understand”**								.03
	Strongly agree	5 (17)		31 (43)				
	Agree	10 (33)		24 (33)				
	Somewhat agree	10 (33)		12 (17)				
	Neither agree nor disagree	2 (7)		4 (6)				
	Somewhat disagree	1 (3)		1 (1)				
	Disagree	2 (7)		0 (0)				
“**The system can do everything I would want it to be able to do”**								.04
	Strongly agree	5 (17)		26 (36)				
	Agree	10 (33)		17 (24)				
	Somewhat agree	5 (17)		12 (17)				
	Neither agree nor disagree	1 (3)		7 (10)				
	Somewhat disagree	4 (13)		9 (12)				
	Disagree	3 (10)		1 (1)				
	Strongly disagree	2 (7)		0 (0)				
“**I would use the HelixVM services again”**								.03
	Strongly agree	10 (33)		49 (68)				
	Agree	12 (40)		11 (15)				
	Somewhat agree	3 (10)		6 (8)				
	Neither agree nor disagree	1 (3)		2 (3)				
	Disagree	3 (10)		2 (3)				
	Strongly disagree	1 (3)		2 (3)				

^a^Percentages may not sum up to 100 due to round up of individual percentages.

**Table 3:** Encounter-type–based differences for patient survey questions with significant *P* values (n=102).

Statements in the questionnaire		Encounter type, n (%)^a^			*P* value
		Fast track (n=70)	Virtual visit (n=32)		
“**The platform provides for my healthcare need”**					.03
	Strongly agree	26 (37)	15 (48)		
	Agree	21 (30)	11 (34)		
	Somewhat agree	16 (23)	2 (6)		
	Neither agree nor disagree	4 (6)	0 (0)		
	Somewhat disagree	0 (0)	3 (9)		
	Disagree	2 (3)	0 (0)		
	Strongly disagree	1 (1)	1 (3)		
“**I can hear the clinician clearly using the telehealth system”**					.01
	Strongly agree	22 (31)	21 (66)		
	Agree	25 (36)	9 (28)		
	Somewhat agree	2 (3)	2 (6)		
	Neither agree nor disagree	17 (24)	0 (0)		
	Somewhat disagree	1 (1)	0 (0)		
	Disagree	2 (3)	0 (0)		
	Strongly disagree	1 (1)	0 (0)		
“**I find the Fasttrack Rx (ability to receive prescription without virtual visit) service useful”**					.03
	Strongly agree	47 (67)	20 (62)		
	Agree	15 (21)	4 (12)		
	Somewhat agree	4 (6)	1 (3)		
	Neither agree nor disagree	0 (0)	5 (16)		
	Somewhat disagree	1 (1)	0 (0)		
	Disagree	1 (1)	0 (0)		
	Strongly disagree	2 (3)	2 (6)		

^a^Percentages may not add up to a 100 due to round up of individual percentage.

**Table 4:** State-based differences for patient survey questions with significant *P* values (n=102).

Statements or questions			State (region), n (%)^a^									*P* value	
			Northeast (n=12)		South (n=69)		Midwest (n=11)		West (n=10)				
“**The way I interact with this system is pleasant”**													*.*002
	Strongly agree	1 (8)		27 (39)		2 (18)		5 (50)					
	Agree	4 (33)		24 (35)		6 (55)		3 (30)					
	Somewhat agree	3 (25)		11 (16)		1 (9)		1 (10)					
	Neither agree nor disagree	0 (0)		6 (9)		1 (9)		0 (0)					
	Somewhat disagree	3 (25)		1 (1)		0 (0)		0 (0)					
	Disagree	1 (8)		0 (0)		0 (0)		1 (10)					
	Strongly disagree	0 (0)		0 (0)		1 (9)		0 (0)					
“**The system can do everything I would want it to be able to do”**													.047
	Strongly agree	3 (25)		21 (30)		3 (27)		4 (40)					
	Agree	2 (17)		22 (32)		2 (18)		1 (10)					
	Somewhat agree	2 (17)		11 (16)		3 (27)		1 (10)					
	Neither agree nor disagree	1 (8)		4 (6)		2 (18)		1 (10)					
	Somewhat disagree	1 (8)		10 (14)		0 (0)		2 (20)					
	Disagree	3 (25)		0 (0)		0 (0)		1 (10)					
	Strongly disagree	0 (0)		1 (1)		1 (9)		0 (0)					

^a^Percentages may not add up to a 100 due to round up of individual percentage.

#### Providers (Phase 1 and Phase 2)

A total of 12 providers successfully completed the web-based survey (phase 1). Of these 12 providers, 10 (91%) completed the in-depth, 1-on-1 interviews (phase 2). Their demographic details are presented in Tables 5 and 6. As the sample size is <30, the demographic variables are presented only in categorical form.

**Figure 2:**
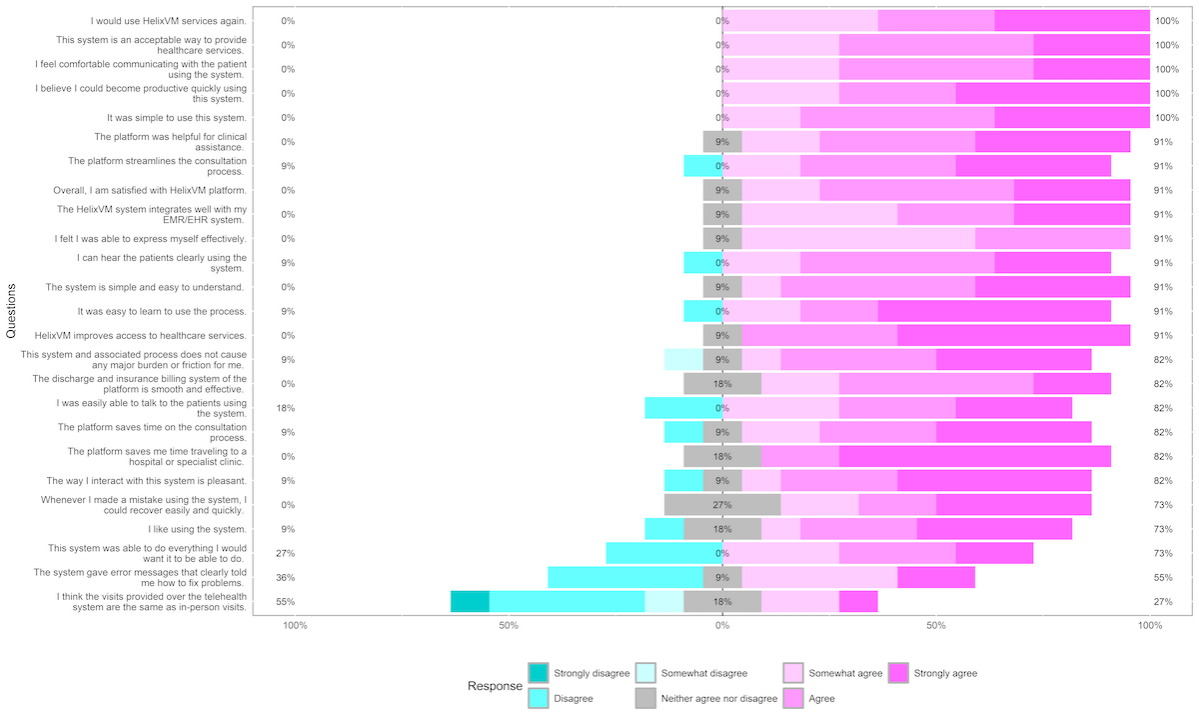
Percentage of responses from the provider survey questionnaire. EHR: electronic health record; EMR: electronic medical record.

Overall, 92% (11/12) of the providers felt that the platform improves access to health care services, it streamlines the consultation process, it was easy to learn to use the process, it was easy to understand, it integrates well with their EMR, and it was helpful for clinical assistance. Moreover, 92% (11/12) of the providers expressed overall satisfaction with HelixVM (Figure 2 contains all questions and response percentages). Approximately 83% (10/12) of the providers felt that they could save time traveling to a clinic or hospital and also save time on the consultation process. All providers (12/12, 100%) found the platform simple to use; furthermore, they felt that they could become productive quickly using this system, that this was an acceptable way to provide health care services, and that they would use HelixVM again. Approximately 75% (9/12) of the providers felt that whenever they made a mistake using the platform, they could recover quickly and easily, whereas 58% (7/12) felt that the system gave error messages that clearly told them how to fix the problems. Approximately 83% (10/12) of the providers felt that the discharge and insurance billing systems were smooth and effective, and approximately 25% (3/12) felt that the virtual visits were the same as in-person visits.

**Table 5:** Provider demographics (survey; n=12).

Characteristic			Participant
**Sex, n (%)**			
	Male	5 (45)	
	Female	6 (55)	
**Age group (y), n (%)**			
	25 to <45	7 (64)	
	≥45 to <75	4 (36)	

**Table 6:** Provider demographics (interviews; n=10).

Provider ID	Sex	Age (y)	Chief complaint^a^
HD1	Male	59	Medication refills and cold and influenza
HD2	Female	29	Cold and influenza and medication refills
HD3	Female	67	Workers’ compensation
HD5	Male	36	Medication refill and UTI^b^
HD7	Male	35	Cold and influenza and medication refill
HD9	Male	44	Prescription refill and cold and influenza
HD10	Female	36	Cold and influenza and UTI
HD13	Female	43	Cold and influenza and UTI
HD14	Female	42	UTI and workers’ compensation
HD15	Male	35	Cold and influenza and prescription or medication refills

^a^The medical condition or ailment for which the patient contacted the provider through the HelixVM marketplace platform.

^b^UTI: urinary tract infection.

### Exploratory Factor Analysis

We derived 2 latent factors based on the eigenvalue criteria of >1 and the scree plot results [16,17] (Multimedia Appendix 5). The factor loadings and statements included in the factors are presented in detail in Table 7. Given the sample size, factor loadings ≥0.5 are highlighted. Factor 1 (F1) consists of >50% of the statements in the questionnaire. These pertain to meeting health care needs and expectations, the simplicity of use and likability when interacting with the system, error recovery, the usefulness of the FastTrack Rx feature, and overall satisfaction. Factor 2 (F2) forms a construct involving questions pertaining to health care access, the acceptability of the platform in receiving health care, meeting health care needs, saving time, likability toward using the system, aspects of the clinician-patient interaction, and overall satisfaction. Qualitatively speaking, F1 asserts the ability of the majority of questions to measure the intended outcomes of user experience, usability, and effectiveness, whereas F2 is more to do with finding telemedicine to be an acceptable way of receiving health care and the quality of the patient-provider digital interaction. Qualitatively speaking, F2 measures the usefulness and the quality of the telemedicine platform. F1 accounts for 36.5% of the variance in the data, while F2 accounts for 32.1%. Cumulatively, both factors explain 68.5% of the variance in the data.

**Table 7:** Factor loadings from exploratory factor analysis using polychoric correlations, minimum residual estimation method for factor loadings, and varimax rotations.

Statements in the modified Telemedicine Usability Questionnaire	Factor 1	Factor 2
“HelixVM improves my access to healthcare services”	0.4930	0.5550
“The platform saved me time from travelling to a doctor, hospital or specialist clinic”	0.4940	0.5231
“The platform provides for my healthcare need”	0.6160	0.5970
“It was simple to use this system”	0.8150	0.4225
“It was easy to learn to use the system”	0.8610	0.2300
“I believe I could receive the care needed quickly”	0.6670	0.5300
“The way I interact with the system is pleasant”	0.7550	0.4880
“I like using the system”	0.7330	0.5310
“The system is simple and easy to understand”	0.8330	0.3828
“This system can do everything I would want it to be able to do”	0.8100	0.3300
“I can easily talk to the clinician and/or receive treatment using this system”	0.6070	0.6200
“I can hear the clinician clearly using the telehealth system”	0.1510	0.7040
“I felt I was able to express myself effectively”	0.3510	0.7580
“Using the system I can see the clinician as well as if we met in person”	0.3066	0.7270
“The system gave error messages that clearly told me how to fix problems”	0.4920	0.1290
“Whenever I made a mistake using the system, I could recover easily and quickly”	0.7060	0.3438
“I find the FastTrack Rx (ability to receive prescription without virtual visit) service useful”	0.5180	0.6170
“I feel comfortable communicating with the clinician using this system”	0.2290	0.7930
“This app/platform is an acceptable way to receive healthcare services”	0.4540	0.7198
“I would use the HelixVM services again”	0.4945	0.6780
“Overall, I am satisfied with HelixVM”	0.6010	0.6390

The results of the analysis, including demographic data (age group, sex, and encounter type), revealed a higher propensity among middle-aged individuals (those aged 30 to <45 y) to rate the platform more positively than the others. Compared to men, women were more likely to rate the platform positively, and individuals who had a virtual visit encounter type were more likely to rate the platform highly than those who had a FastTrack Rx encounter type. Individuals who rated themselves between 5 and 7 (medium level) on technology savviness tended to rate the platform more positively. Furthermore, individuals who resided in the South region of the United States rated the platform more positively. For all such individuals, the preference was for all aspects collected in the survey (F1), especially those aspects related to saving time, health care services, and interaction with clinicians (F2). The biplots for sex, age group, encounter type, technology savviness, and state are presented in Multimedia Appendix 6. These biplots display the relationships between the factor items (survey questions) and factor scores for 2 factors: F1 (MR1) and F2 (MR2). The clustering of factor items toward the top right of the charts for the different groups such as female sex, virtual visit encounter type, age group 30 to <45 years, high level of technology savviness, and southern US states evidences the high propensity among these demographic subgroups to rate the platform highly (ie, more positively).

### Provider Interview Analysis

#### Overview

Overall, 15 main questions, covering various themes, were asked to the providers. These themes were further refined based on any underlying messages identified from the responses. The different themes and their findings are detailed herein.

**Figure 3:**
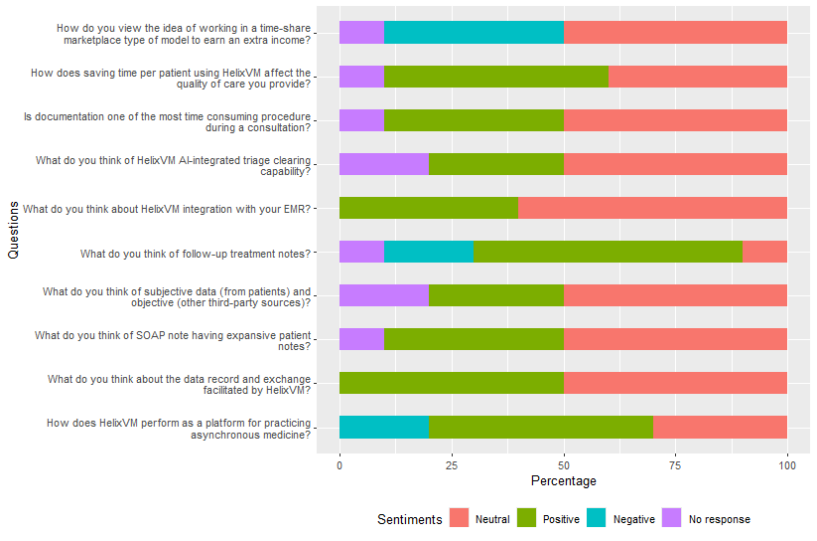
Sentiment analysis of provider interview responses. AI: artificial intelligence; EMR: electronic medical record; SOAP: subjective, objective, assessment, and plan.

**Figure 4:**
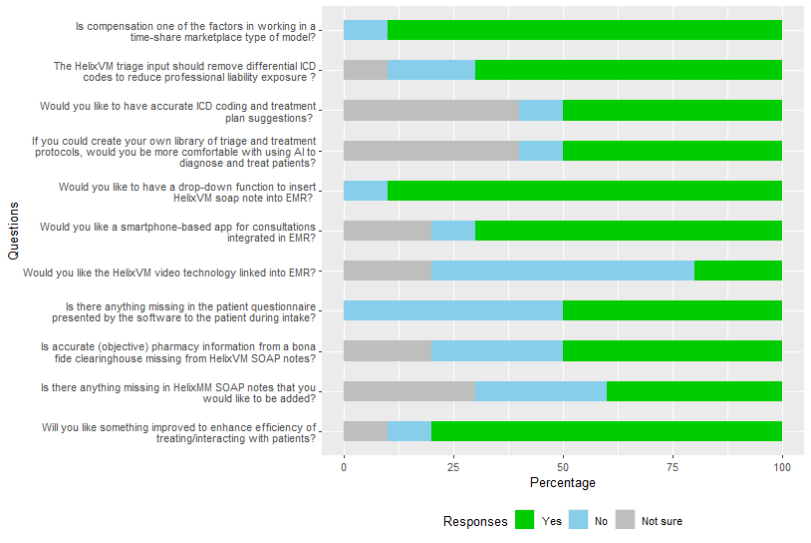
Percentage of categorical responses from the provider interviews. AI: artificial intelligence; EMR: electronic medical record; ICD: International Classification of Diseases; SOAP: subjective, objective, assessment, and plan.

**Figure 5:**
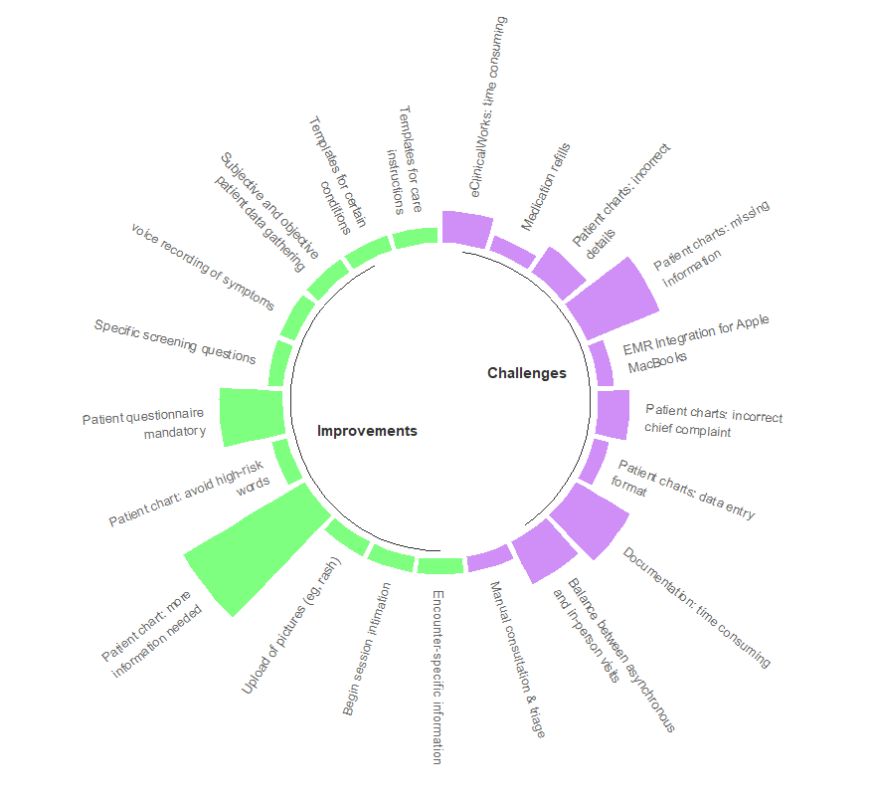
Challenges faced by the providers and suggestions for improvements to the platform. EMR: electronic medical record.

For all qualitative responses from providers, we interpreted the sentiments as positive, neutral, or negative. The results are presented in Figure 3. All categorical responses to the interview questions are summarized in Figure 4. In addition, we segregated the interview responses to identify the challenges faced by the providers and their suggestions for improvements to the platform, as illustrated in Figure 5.

#### Thematic and Sentiment Analysis

##### Asynchronous Medicine: Accessibility and Quality of Care

Providers acknowledged the potential of asynchronous medicine to improve patient accessibility and convenience, particularly for medication refills, minor illnesses, and scheduling flexibility. This could enhance the overall patient experience by breaking down barriers to care. However, they viewed asynchronous medicine as a valuable supplement rather than a replacement for traditional in-person care. While recognizing its advantages, the providers noted limitations in fully assessing patients, especially those requiring physical examinations. Proper screening and filtering were emphasized to ensure safety and avoid misdiagnosis. Committed to high standards of care, providers aimed to balance leveraging asynchronous medicine’s benefits with upholding quality and patient safety. Our findings showed that providers view this technology as a tool to increase health care accessibility and simplify procedures, particularly for patients with busy schedules or limited access to traditional health care facilities.

However, opinions varied regarding the effectiveness of asynchronous medicine. While some of the providers saw its value in specific situations, others had concerns about its limitations, such as the possibility of missing important information and the inability to pick up on visual cues or environmental factors. The providers emphasized that the effectiveness of asynchronous visits in enhancing patient outcomes relied greatly on the quality and thoroughness of information provided by patients, the effectiveness of prioritizing and gathering standardized information, and the capability to uphold meaningful communication during remote consultations. Despite the perks of asynchronous medicine, the providers highlighted the importance of adopting a balanced approach that places emphasis on comprehensive data, efficient prioritization, and meaningful communication to attain enhanced patient outcomes.

Providers laid strong emphasis on the importance of proper screening and filtering of patients to ensure their safety and to avoid over- or undertreatment. The providers are committed to maintaining high standards of care by making the best decisions possible within the constraints of asynchronous medicine and offering alternative options when needed. Thus, they strived to strike a balance between leveraging the benefits of asynchronous medicine and ensuring that quality standards and patient safety are upheld in health care delivery. This diversity in viewpoints highlights the importance of carefully weighing the pros and cons of asynchronous medicine, stressing the need for thorough evaluation, and addressing any potential risks to patient safety. The following quote exemplifies this balanced view and the recognition of the benefits of asynchronous medicine:

I think it’s a good idea as far as accessibility goes. It definitely makes it easier for people to kinda get in and get out and potentially get medications or receive medical recommendations and things like that. I definitely don’t think that the person aspect should be completely taken out of medicine. Just because I think like, writing your whatever is going on with you into a computer and then just having it spat out on the other side and just read by somebody I sometimes don’t think it’s enough. Umm, just because you do have to have those follow-up questions and but in some circumstances asynchronous is a really good tool.

HD2

##### Time and Productivity

Providers must allocate sufficient time to understand the patient’s medical history, current symptoms, and concerns. This initial investment in time lays the foundation for accurate diagnosis and effective treatment planning. Rushing through this phase can lead to oversights, misdiagnoses, or inadequate treatment strategies. However, excessive time spent on 1 consultation can disrupt the flow of appointments, causing delays and impacting overall productivity. Providers often find themselves juggling the need to allocate adequate time for each patient while ensuring that the clinic operates smoothly and avoids prolonged waiting times. Productivity in medical consultations is measured not only by the number of patients seen but also by the quality of care delivered within a reasonable time frame.

Our interviews with providers shed light on the challenges they faced in this context. Key challenges included difficulties in the consultation process, particularly in medication qualification, maintaining patient focus, and ensuring the efficient use of EMR systems. Qualifying patients for medications such as for weight loss or diabetes management involves extensive paperwork and insurance authorization, which adds complexity to the process. In addition, patients often veer off topic during consultations, necessitating frequent redirection. This further adds to the provider time spent on a consultation. Cumbersome EMR systems, difficulty in obtaining complete patient information, and connectivity issues further prolong consultations thereby impacting efficiency:

I would definitely say patients that don’t provide enough information and hard to get in contact with to get the information that’s needed...=triage information that’s missing history and things like that. So it takes a little more time to get in contact with them and actually get all of their background in the chart prior to actually getting a diagnosis and treatment.

HD10

To help address some of these challenges, the providers identified some key improvement areas such as streamlining medication qualification processes, enhancing patient focus during consultations, and optimizing EMR efficiency. These challenges underscore the need for ongoing refinement in telemedicine platforms such as HelixVM to address issues that affect provider productivity and patient care quality.

Documentation was another key aspect of asynchronous medicine that the providers viewed as time consuming. These perspectives varied because although documentation can be time consuming, it is still critical for effective care process. While some of the providers faced challenges in gathering the necessary documentation, others emphasized its critical role in providing comprehensive care and ensuring smooth visit encounters. Positive sentiments were expressed toward the current documentation platform, despite acknowledging its learning curve and variability in documentation time across different patient cases. In general, despite facing challenges, documentation was widely acknowledged as essential for effective communication and decision-making among providers. However, there is a clear need for improvement, as shown by the sentiment analysis: 50% (5/10) of the providers were neutral toward the documentation process in HelixVM, and only 30% (3/10) expressed positive emotions.

##### Integration Within Workflow

The integration of HelixVM with providers’ existing workflow of processes and systems received predominantly positive feedback. HelixVM seamlessly integrates with the providers’ EMR systems. This is evident from the sentiment analysis of responses, which showed that 60% (6/10) of the providers are content with how HelixVM fits into their EMR setup. On the flip side, only 20% (2/10) had negative feedback, but, overall, it seems that most providers (6/10, 60%) are satisfied with this integration. However, it is essential to acknowledge that while many users praised the integration’s efficiency, some encountered challenges related to the EMR platform itself. This suggests that providers’ experiences and perspectives on EMRs could impact their workflow when integrating HelixVM. The following quote exemplifies the providers’ satisfaction and comfort with how HelixVM has integrated with their EMR:

I think it has been very, very smooth considering the vast amount of information that we’ve had to use and how easily it’s been able to, umm, interface. I really don’t see any major difficulties in it. We basically use the same system that we use in our urgent care brick-and-mortar setting as we do with telemedicine and it’s been like that from the beginning. Thank God it’s we have had very few glitches in terms of the system. It’s only been getting better, yes.

HD1

Feedback on documentation integration varied. While some users found the current system effective, others identified areas for improvement. Suggestions included condition-specific surveys, more thorough current illness histories, stricter documentation standards, mandatory field completion, and a patient feedback feature. These recommendations aimed to enhance documentation specificity, completeness, and the ease of use.

The providers also raised concerns about workflow integration with HelixVM. They highlighted the need for better out-of-state prescription processing and faster system performance, especially with eClinicalWorks (a widely used EMR and practice management software system). In addition, suggestions included ensuring complete patient intake information and encouraging thorough patient input. Overall, our interviews complemented the survey results. Providers generally approved of the documentation platform while acknowledging the learning curve and ongoing improvements. They recognized that documentation time varies depending on the case, with some encounters requiring more detailed charting for treatment plans and care continuity.

##### Data Exchange

HelixVM’s data exchange platform proves its usefulness by enabling efficient communication and follow-up, according to the providers. However, a recurring issue identified by providers was the presence of discrepancies between medication records and patient-reported information. Challenges with data entry, formatting inconsistencies, and connectivity problems were reported. In addition, providers emphasized the need for more detailed medical histories within the records to improve the platform’s comprehensiveness and aid in making informed decisions:

Another thing that I’ve come across is the chief complaint might be listed as a headache, but then when I read their current problem, they’re talking about something totally different. Not at all about a headache, so I don’t know how that would be corrected other than what I do in that case is I send the chart back to the medical assistant and tell them they need to contact the patient and get more clarification. So, I think that needs a little bit of work.

HD3

The quote by HD3 highlights how sometimes the information listed for the main reason for a patient’s visit does not match what the patient wants to discuss. This makes it hard for the provider to understand the patient’s needs and compels them to spend additional time on clarification. The providers believe that the platform should be improved to avoid this issue. Data security and privacy within HelixVM are a top priority for providers. They stressed the importance of adhering to HIPAA compliance and maintaining robust security measures. Overall, there is a prevailing trust in the system’s ability to safeguard patient information. However, concerns were raised regarding potential security risks associated with remote work environments, such as the use of PCs by employees. Ongoing vigilance and strict adherence to security protocols are recognized as crucial to mitigating these risks. While confidence exists in the platform’s current security measures, continuous education and evaluation are deemed essential to address potential vulnerabilities and ensure the continued protection of patient data.

Sentiment analysis for this theme revealed varying opinions on different aspects of HelixVM use. For data record and exchange, 60% (6/10) of the responses were neutral, indicating a balanced perception of the platform’s usefulness, while 40% (4/10) were positive, reflecting satisfaction with its functionality. The subjective, objective, assessment, and plan (SOAP) notes elicited mixed opinions, with 40% (410) neutral, 50% (5/10) positive, and 10% (1/10) negative responses. Subjective and objective data specifically garnered 50% (5/10) neutral and 40% (4/10) negative feedback, highlighting challenges in obtaining comprehensive patient information. Follow-up treatment notes received 50% (5/10) neutral and 30% (3/10) positive responses, indicating a moderate level of satisfaction with the information provided.

##### AI Triage

A variety of user experiences and perspectives emerged from the research on HelixVM’s AI triage system. Divergent views on the integration and streamlining of the triage process were central to these findings. Approximately 40% (4/10) providers expressed satisfaction with the AI triage system. They praised its ability to facilitate comprehensive care and support medical decision-making. Providers viewed the system as a prime example of technological innovation in health care. However, approximately 60% (6/10) providers indicated areas for improvement. These providers highlighted the need for better system integration and emphasized the importance of patient-provided information in effective triage. Providers acknowledged the evolving nature of AI in medicine, suggesting that its full potential remains to be harnessed. The following quote exemplifies this sentiment:

We can leverage AI as long as it doesn’t replace health care professionals. While it improves efficiency, AI in medicine is still in its early stages and has a lot to learn.

HD14

Sentiment analysis of the interview data for this theme revealed deeper nuances in these perspectives. It showed that 40% (4/10) of the responses conveyed a positive sentiment, signifying satisfaction or appreciation, while 60% (6/10) were neutral, reflecting a mixed or indifferent sentiment. This distribution mirrored the varied views expressed by respondents on the current state of the HelixVM triage system.

The feedback on HelixVM’s AI-integrated triage capabilities was mixed. While some users expressed overall satisfaction, praising its effectiveness in supporting comprehensive care, others indicated that there is still room for improvement, particularly in terms of better integration and functionality. A few users noted that they had limited involvement in the triage process and, therefore, could not provide direct feedback. One user highlighted the significance of patient-supplied information for accurate triage, whereas another appreciated the efficiency that AI brought to the process, albeit recognizing its evolving role in the medical field. In addition, a respondent underscored the practical utility of AI in medical decision-making.

Several providers suggested enhancements to the system, including broadening the scope of patient concerns, improving the functionality for ordering laboratory tests and imaging, and developing mechanisms to better detect missing patient information to avoid incomplete records. Some users also raised concerns regarding patient behavior, such as skipping questions or providing incomplete information, emphasizing the need for systems to mitigate these challenges. Overall, while the AI triage system was considered functional, there was a consensus that further refinements are necessary to enhance its comprehensiveness and efficiency in supporting patient care.

This study also demonstrates the confirmability, dependability, credibility, and transferability of the thematic analysis. For confirmability, we cross-verified responses from the survey and interviews where the underlying theme of the questions was the same or similar and where the responses to interview questions were similar in theme to the statements in the survey questionnaire; for instance, if participants strongly agreed with the survey statement “This system is an acceptable way to provide healthcare services,” we compared their responses to the interview question “What do you think about asynchronous medicine as a way for improving patient access?” The findings were consistent, with respondents expressing optimism about asynchronous medicine as a means for delivering health care, reflecting an overall positive sentiment. Similarly, in the interview, a participant, in response to the aforementioned question, elaborated on the limitations of asynchronous medicine, noting that some symptoms may be difficult to assess compared to an in-person visit, while acknowledging it to be a useful tool. This participant answered “*somewhat agree*” to the corresponding survey question. The same participant answered “*strongly disagree“* to the survey statement “I think the visits provided over the telehealth system are the same as in-person visits,” thus maintaining consistency between their survey and in-depth interview responses. Participants who answered “*strongly agree* to the survey statement “I believe I could become productive quickly using this system” responded in the affirmative when asked in the interview “Do you think saving time per patient using HelixVM can contribute significantly to increasing your productivity?”

To aid the dependability of our study, we documented every aspect of the process, including participation recruitment, onboarding, compensation, survey administration, survey questionnaires, interview scripts, and the interview guide, as well as the methodologies used for both qualitative and quantitative analyses.

To establish the credibility of our study, we conducted each interview for at least 60 minutes, discussing all 15 questions in detail. When needed, we extended the interview duration (with the participant’s consent) to gather detailed responses for all questions. We have provided rich accounts of the providers’ responses and highlighted the validation of study results by comparing the responses from both the survey and interviews.

The findings from the thematic analysis are transferable to providers who use telemedicine as part of their practice. The provider interview guide contains questions designed to elicit insights into providers’ overall experiences with telemedicine and its functionality. While these findings may be generalized to other providers in similar telemedicine settings, those specific to HelixVM should not be considered generalizable.

### Challenges and Suggestions for Improvement

The providers highlighted some of the challenges that they faced. Most of them cited issues with patient charts. One issue was that the charts reach the provider even if the patients fill them out incorrectly, or they have missing information; for example, patient charts requesting prescription refills may lack essential dosage information, but they still reach the provider. Similarly, the chief complaint might be listed as a “headache,” but upon reviewing the current problem, the provider discovers that the patient is talking about a completely different issue. This necessitates sending the chart back to the medical assistant to contact the patient for clarification. All this adds to provider time spent on the process and does not make for a successful telehealth encounter.

An issue with the current system is that it does not allow patients to select certain symptoms owing to the data entry format, compelling them to type these in manually. The system also does not present information relevant to a visit, such as certain symptoms that may be indicative of various conditions. Challenges in data security and safety arise concerning specific needs for asynchronous medication refills, especially for out-of-state patients. Most providers resort to using PCs, which poses a risk because not everyone is equipped with the knowledge to securely handle sensitive information and other details. A few providers noted that using the eClinicalWorks platform was difficult and time consuming. The time-consuming nature of the documentation process was also highlighted by a provider.

The providers offered several suggestions to help improve the productivity and efficiency of the HelixVM platform. To ensure that patient charts are complete and useful, most of the providers suggested making it mandatory for patients to answer all questions in the patient questionnaire before proceeding on the platform. Furthermore, the questionnaire should allow for subjective and objective gathering of patient data, which could be specific for a given condition. This process could be enhanced by allowing patients to upload pictures. These could be of the symptoms they are experiencing, such as a rash or any other ailment where applicable. A voice-to-text or voice-recording feature could be included for patients to voice record their symptoms, which would then be directly captured in their charts. Specific screening questions tailored to each complaint could be added to enhance the thoroughness of the patient charts. Furthermore, users who do not require medical attention could be filtered out at the beginning of the process to prevent wasting provider time. This is particularly relevant for patients requesting controlled substances such as hydrocodone or Xanax. Another category of patients who should not access the platform includes those who use high-risk words such as “chest pain,” “choking,” or “breathing issues.” Such patients should be redirected to the emergency department. In addition, some patients may misinterpret the term “chest pain” to refer to discomfort from a cough. Therefore, the use of such high-risk words should be avoided in the SOAP notes. For providers, the SOAP notes could include templates tailored to specific conditions or care instructions.

To save time, providers suggested implementing a mechanism to notify them when the patient shows up in the “room.” Sometimes, providers wait 5 to 10 minutes for patients to join the consultation. There should be increased engagement and participation of patients regarding referrals to specialists or for diagnostic tests. Systems should be established to encourage providers to document all relevant information, including their thoughts when making a diagnosis and the reasoning behind it. This would be useful for another provider treating the same patient and also strengthen and support the diagnosis. Furthermore, this information could be useful for patients. Finally, the platform could include features for ordering laboratory tests, such as imaging and other diagnostics.

## Discussion

### Principal Findings

We report on the findings of a mixed methods study on the usability and effectiveness of a telemedicine platform as assessed by patients and health care providers. Overall, 86% (88/102) of the patients reported high levels of satisfaction and ease of use with the HelixVM marketplace platform. The patients rated the platform highly for the simplicity of use (92/102, 90%) and for interactions with clinicians (89/102, 87% to 91/102, 89%). They also rated the system highly for the technical quality of the patient-clinician interaction (77/102, 75% to 81/102, 79%). In addition, the ability to recover from mistakes made by patients when using the system received a positive response (78/102, 76%).

Importantly, the patients agreed that using a platform such as HelixVM saves them time (91/102, 89%), and they found the fast-track service for receiving a prescription without a virtual visit to be helpful (91/102, 89%). The differences in responses across all levels of agreement and disagreement for each question in the patient survey are statistically significant (*P*<.01). As the provider dataset had <30 participants, *P* value analysis was not possible. Factor analysis of the patient survey response data revealed latent demographical patterns related to sex, age group, encounter type, US state, and technology savviness. Individuals who rated the platform higher tended to be women aged 30 to <45 years, those who had a virtual visit encounter type, those with medium levels of technology savviness, and those residing in the South region of the United States. These demographic patterns also met statistical significance for sex and encounter type for some of the questions (Tables 2-4).

Sex-based, within-group differences were statistically significant for questions related to the ease of learning and understanding the system, agreeing that the system can do everything that they would want it to do, and, overall, using the services again. Women rated the platform higher than men (Table 2). For encounter-based, within-group differences in responses, the key question on the usefulness of the fast-track prescription service showed statistically significant differences (Table 3) For state-based, within-group differences, the responses to the question on “pleasant interaction with the system” were statistically significant (Table 4).

Qualitatively speaking, women, especially those aged 30 to <45 years, tend to juggle multiple responsibilities such as managing housework, children, jobs, and so on, and thus find it convenient to access health care through telemedicine. They find it easier to learn and understand how to use telemedicine platforms, making the format more appealing for them to use again. Individuals who had the virtual visit encounter type would have used the platform’s full range of services and thus would be able to appreciate the platform’s performance and its ability to meet their needs.

For state-based differences, one possible explanation could be that the HelixVM platform first began operations in Florida and then expanded to other states. It is possible that it has a high patient base in Florida and that most providers too are based in Florida.

The providers in our study emphasized the importance of careful screening and the potential risks of missing crucial information during telehealth encounters

### Comparison to Previous Studies

#### Overview

The literature on the use of telemedicine services is not comprehensive and mostly centers around the adoption of telemedicine during the COVID-19 pandemic and its efficiency in delivering health care. This may be mostly because of the low adoption of telemedicine before the pandemic, while during the pandemic, the adoption rates soared almost 3-fold [5]. Even otherwise, most studies focus on telehealth interventions, remote monitoring interventions, the use of SMS text messaging, and the use of videoconferencing. There are limited studies on web-based telemedicine platforms, and where such studies exist, the sample sizes are small in comparison to those in studies on other technologies [18,19].

#### Patients

Overall, our findings on patient survey responses agree with the findings from other studies on reporting high patient satisfaction with the flexibility and time-saving aspects of HelixVM [18,20]. Patient satisfaction has been identified as a growing concern in health care [19]. It is especially important for telemedicine where the reliance on quality of the health care received is evaluated solely based on patient feedback [19]. The patient’s satisfaction was on account of easy access, the quality of care, convenience, and the understanding of telehealth [18,21].

Our findings regarding the higher use of telemedicine by women and younger age groups are consistent with those of other studies [22]. In addition, the preference for telehealth visits among women compared to men aligns with existing research [21]. Other studies have reported improved behavioral outcomes for women using web-based telemedicine interventions [18].

#### Providers

Our findings on asynchronous medicine and telemedicine are in agreement with those of other studies that show that providers find asynchronous medicine valuable for improving access to care, particularly for the convenient management of simple cases or for patients facing transportation barriers [2,23]. However, our research also echoes concerns raised in prior studies [24,25] regarding limitations in fully assessing patients asynchronously. The providers in our study emphasized the importance of careful screening and the potential risks of missing crucial information during telehealth encounters. This reinforces the need for the ongoing development of clear protocols and best practices for asynchronous consultations.

While some of the providers expressed satisfaction with the platform’s integration with existing workflows and EMR systems, others highlighted challenges, indicating the need for further integration and customization. This aligns with the broader literature on telemedicine integration, which emphasizes the importance of seamless data exchange and clear communication protocols [26]. Finally, the mixed user experiences regarding the AI triage system demonstrate the need for ongoing development and user education in such telemedicine systems. This aligns with other studies that emphasize the importance of continuous improvement alongside user education for the successful implementation of telemedicine with AI components [27]. Providers who found the AI triage system helpful appreciated its potential for efficiency and comprehensive care. However, others highlighted the limitations of the system and the importance of patient-provided information. This suggests that HelixVM’s AI triage system should continue to be developed and refined, while ensuring clear communication about its role and limitations to both patients and providers.

### Strengths and Limitations

The mixed methods study design involving the same population is a key strength of our study. Administering the modified version of the TUQ in phase 1 and then using the responses to guide the structured interviews in phase 2 helped us to dive deeper to gain an understanding of the providers’ perspectives on the various value propositions of the HelixVM platform. Interviewing the providers after the surveys helped us to explore several nuances that otherwise would not have been captured by the survey alone. The demonstrated confirmability, credibility, dependability, and transferability highlight the strengths of the thematic analysis performed in this study. The methods used to analyze patient survey responses met statistical standards satisfaction due to the substantial sample size (n=102), making it suitable for testing statistical significance. The wide range and number of questions (22) asked to the patients allowed us to perform factor analysis, an exploratory statistical technique, to reveal latent patterns confirmed statistically. The survey questions for both the patients and providers were specific to their hands-on experiences of using the HelixVM marketplace platform, ensuring that the findings reflect real-life experiences. Of note, even for the older patient population, the use of HelixVM and the completion of the survey did not require assistance. This means that there was no prompting or suggestive bias in their responses to the questions. The questionnaire design was specific to the use of HelixVM, which allowed us to capture the digital health experience related to HelixVM alone rather than telemedicine in general. Although the provider interviews touched upon a general perspective on asynchronous medicine, compensations, and main practice, these responses were to questions which were separate from specific questions about HelixVM.

Our study has certain limitations too. First, the modified version of the TUQ consisted of questions that required the participants to recall from memory the errors encountered, resolutions provided, and technical glitches. Participants may have difficulty in remembering these details, which is likely to introduce recall bias. This limitation cannot be addressed by the analysis. Participants may also tend to recall those incidents that were memorable. This recall bias can lead to either underestimation or overestimation of the issues encountered. However, regardless of the direction of the bias, its effect will be systematic, meaning that it will not impact the relative findings and patterns reported in this study

Second, the patient population was drawn from 33 US states, with ages ranging from 18 to 65 years. Although we acknowledge that a more balanced distribution of data across the different age groups and US states could enhance statistical stability, this is not strictly a limitation. The study was originally designed to recruit between 100 and 120 patients, and the sampling frame was expanded only to improve the rate of patient recruitment, given the time-sensitive nature of this study. Thus, the high *nonresponse* rate of the patients is not a strict limitation because recruitment was conducted on a first-come basis, prioritizing those who completed participation first.

The third limitation is the small provider population (n=11) in the phase 1 survey, which renders it unfavorable for statistical analysis. One reason for this was the smaller (compared to the patients) sampling frame provided by HelixVM. It is also expected that providers will be outnumbered by patients in a study sample, given the physician-to-patient ratio in health care. Despite efforts to improve recruitment through multiple follow-ups and reminders, we were not able to increase the participation rate further. Fourth and last, we faced limitations in time and resources, which restricted our ability to conduct in-depth interviews with our patient population.

### Recommendations for Future Studies

Given the interesting findings of our study and the strengths and limitations, future studies may be planned to investigate the usability and effectiveness of HelixVM once the suggested improvements are incorporated by the platform. Survey-based studies on providers may be planned, and these should include a larger sample size (n>30) to allow for statistical analysis. Purposive sampling with a good representation across the different age groups and geographic boundaries (ie, covering as many US states as possible) is recommended. From the providers’ perspectives, future studies on the development of protocols and best practices for asynchronous consultations should be of high importance.

For patient-centric future studies, quantitative studies recruiting patients across more US states and with a sufficient sample size for demographic subgroups may be planned. Furthermore, qualitative studies on patients may also be planned, with 1-on-1 interviews to help understand the behavioral nuances of patients who reportedly skip filling out important information and rush through the process to reach the consultation stage or to obtain prescriptions. This lack of completed information was highlighted as a key factor leading to longer consultation times. Future studies may also focus on this aspect to enhance the platform’s functionality, thereby maintaining and preferably increasing patient uptake. Future studies collecting detailed demographic data on ethnicity, geographic location, and type of residence (urban or rural) will provide more insight into the adoption of telemedicine in general and that of HelixVM in particular. Furthermore, future studies can also explore the impact of HelixVM’s AI triage system and its contributions to the critical and evolving conversation about AI integration in telemedicine. The findings from this study can inform future research and development efforts aimed at optimizing AI’s role in asynchronous health care delivery in this rapidly developing field.

### Conclusions

The findings from this study were positive overall for the HelixVM marketplace platform. Data exchange through HelixVM seems efficient, but concerns exist regarding data gaps and record accuracy. There is a need for the ongoing development of clear protocols and best practices for asynchronous consultations. These findings point to the need for improved data management strategies within the platform. The findings from this study contribute to existing literature on asynchronous medicine in several ways. By focusing on HelixVM, we provide a detailed analysis of a specific platform’s functionalities, its integration with existing workflows, and the user experiences of both patients and providers. This deep dive into a real-world implementation adds valuable depth to the broader literature, which often focuses on theoretical models or pilot studies.

## Appendix

Multimedia Appendix 1 Modified Telehealth Usability Questionnaires for patients and providers.

Multimedia Appendix 2 Provider interview questions guide.

Multimedia Appendix 3 Cronbach α values.

Multimedia Appendix 4 Goodness-of-fit measure (*P* values).

Multimedia Appendix 5 Scree plot.

Multimedia Appendix 6 The biplots for sex, age group, encounter type, technology savviness, and US state.

## Abbreviations

AI: artificial intelligence
EMR: electronic medical record
HIPAA: Health Insurance Portability and Accountability Act
HITLAB: Healthcare Innovation and Technology Lab
SOAP: subjective, objective, assessment, and plan
TUQ: Telemedicine Usability Questionnaire

## Data Availability

The datasets generated and analyzed during this study are available from the corresponding author on reasonable request.
