# Supplementary material for: Assessing the Usability and Effectiveness of an AI-Powered Telehealth Platform: Mixed Methods Study on the Perspectives of Patients and Providers
Source: JMIR Form Res. 2024 Nov 25;8:e62742. doi: 10.2196/62742 (PMC11629036; doi:10.2196/62742)
Supplement: Multimedia Appendix 1 [file formative_v8i1e62742_app1.docx]

### QUESTIONNAIRE 1 FOR PATIENTS

| **#** | **Statements** | **N/A** | **1 2 3 4 5 6 7** |
| --- | --- | --- | --- |
| 1 | HelixVM improves my access to healthcare services. | ☐ | DISAGREE ☐ ☐ ☐ ☐ ☐ ☐ ☐ AGREE |
| 2 | The platform saved me time traveling to a doctor, hospital or a specialist clinic. | ☐ | DISAGREE ☐ ☐ ☐ ☐ ☐ ☐ ☐ AGREE |
| 3 | The platform provides for my healthcare need. | ☐ | DISAGREE ☐ ☐ ☐ ☐ ☐ ☐ ☐ AGREE |
| 4 | It was simple to use this system. | ☐ | DISAGREE ☐ ☐ ☐ ☐ ☐ ☐ ☐ AGREE |
| 5 | It was easy to learn to use the system. | ☐ | DISAGREE ☐ ☐ ☐ ☐ ☐ ☐ ☐ AGREE |
| 6 | I believe I could receive the care I needed quickly using this system. | ☐ | DISAGREE ☐ ☐ ☐ ☐ ☐ ☐ ☐ AGREE |
| 7 | The way I interact with this system is pleasant. | ☐ | DISAGREE ☐ ☐ ☐ ☐ ☐ ☐ ☐ AGREE |
| 8 | I like using the system. | ☐ | DISAGREE ☐ ☐ ☐ ☐ ☐ ☐ ☐ AGREE |
| 9 | The system is simple and easy to  understand. | ☐ | DISAGREE ☐ ☐ ☐ ☐ ☐ ☐ ☐ AGREE |
| 10 | This system can do everything I would want it to be able to do. | ☐ | DISAGREE ☐ ☐ ☐ ☐ ☐ ☐ ☐ AGREE |
| 11 | I can easily talk to the clinician and/or receive treatment using this system. | ☐ | DISAGREE ☐ ☐ ☐ ☐ ☐ ☐ ☐ AGREE |
| 12 | I can hear the clinician clearly using the telehealth system. | ☐ | DISAGREE ☐ ☐ ☐ ☐ ☐ ☐ ☐ AGREE |
| 13 | I felt I was able to express myself effectively. | ☐ | DISAGREE ☐ ☐ ☐ ☐ ☐ ☐ ☐ AGREE |
| 14 | Using the system, I can see the clinician as well as if we met in person. | ☐ | DISAGREE ☐ ☐ ☐ ☐ ☐ ☐ ☐ AGREE |
| 15 | I find the Fast-track Rx (ability to receive prescription without virtual visit) service useful. | ☐ | DISAGREE ☐ ☐ ☐ ☐ ☐ ☐ ☐ AGREE |
| 16 | Whenever I made a mistake using the system, I could recover easily and quickly. | ☐ | DISAGREE ☐ ☐ ☐ ☐ ☐ ☐ ☐ AGREE |
| 17 | The system gave error messages that clearly told me how to fix problems. | ☐ | DISAGREE ☐ ☐ ☐ ☐ ☐ ☐ ☐ AGREE |
| 18 | I feel comfortable communicating with the clinician using the system. | ☐ | DISAGREE ☐ ☐ ☐ ☐ ☐ ☐ ☐ AGREE |
| 19 | This app/platform is an acceptable way to receive healthcare services. | ☐ | DISAGREE ☐ ☐ ☐ ☐ ☐ ☐ ☐ AGREE |
| 20 | I would use the HelixVM services again. | ☐ | DISAGREE ☐ ☐ ☐ ☐ ☐ ☐ ☐ AGREE |
| 21 | Overall, I am satisfied with HelixVM | ☐ | DISAGREE ☐ ☐ ☐ ☐ ☐ ☐ ☐ AGREE |
| 22 | On a scale of 1 to 10, how would you rate yourself on tech savviness. | - | 10 – high tech savviness; 0 – not at all tech savvy |

**In this questionnaire, 1 - strongly disagree, 2 – disagree, 3 – somewhat disagree, 4 – neither**

**agree nor disagree, 5 – somewhat agree, 6 – agree, 7 – strongly agree**

### QUESTIONNAIRE 2 FOR PROVIDERS

| **#** | **Statements** | **N/A** | **1 2 3 4 5 6 7** |
| --- | --- | --- | --- |
| 1 | HelixVM improves access to healthcare services. | ☐ | DISAGREE ☐ ☐ ☐ ☐ ☐ ☐ ☐ AGREE |
| 2 | The platform saves me time traveling to a hospital or specialist clinic. | ☐ | DISAGREE ☐ ☐ ☐ ☐ ☐ ☐ ☐ AGREE |
| 3 | The platform streamlines the consultation process. | ☐ | DISAGREE ☐ ☐ ☐ ☐ ☐ ☐ ☐ AGREE |
| 4 | The platform saves time on the consultation process. | ☐ | DISAGREE ☐ ☐ ☐ ☐ ☐ ☐ ☐ AGREE |
| 5 | The platform was helpful for clinical assistance. | ☐ | DISAGREE ☐ ☐ ☐ ☐ ☐ ☐ ☐ AGREE |
| 6 | It was simple to use this system. | ☐ | DISAGREE ☐ ☐ ☐ ☐ ☐ ☐ ☐ AGREE |
| 7 | It was easy to learn to use the process. | ☐ | DISAGREE ☐ ☐ ☐ ☐ ☐ ☐ ☐ AGREE |
| 8 | I believe I could become productive quickly using this system. | ☐ | DISAGREE ☐ ☐ ☐ ☐ ☐ ☐ ☐ AGREE |
| 9 | The way I interact with this system is pleasant. | ☐ | DISAGREE ☐ ☐ ☐ ☐ ☐ ☐ ☐ AGREE |
| 10 | I like using the system. | ☐ | DISAGREE ☐ ☐ ☐ ☐ ☐ ☐ ☐ AGREE |
| 11 | The system is simple and easy to understand. | ☐ | DISAGREE ☐ ☐ ☐ ☐ ☐ ☐ ☐ AGREE |
| 12 | This system was able to do everything I would want it to be able to do. | ☐ | DISAGREE ☐ ☐ ☐ ☐ ☐ ☐ ☐ AGREE |
| 13 | I was easily able to talk to the patients using the system. | ☐ | DISAGREE ☐ ☐ ☐ ☐ ☐ ☐ ☐ AGREE |
| 14 | I can hear the patients clearly using the system. | ☐ | DISAGREE ☐ ☐ ☐ ☐ ☐ ☐ ☐ AGREE |
| 15 | I felt I was able to express myself effectively. | ☐ | DISAGREE ☐ ☐ ☐ ☐ ☐ ☐ ☐ AGREE |
| 16 | The HelixVM system integrates well with my EMR/EHR system. | ☐ | DISAGREE ☐ ☐ ☐ ☐ ☐ ☐ ☐ AGREE |
| 17 | This system and associated process does not cause any major burden or friction for me. | ☐ | DISAGREE ☐ ☐ ☐ ☐ ☐ ☐ ☐ AGREE |
| 18 | I think the visits provided over the  telehealth system are the same as in-person visits. | ☐ | DISAGREE ☐ ☐ ☐ ☐ ☐ ☐ ☐ AGREE |
| 19 | Whenever I made a mistake using the system, I could recover easily and quickly. | ☐ | DISAGREE ☐ ☐ ☐ ☐ ☐ ☐ ☐ AGREE |
| 20 | The system gave error messages that clearly told me how to fix problems. | ☐ | DISAGREE ☐ ☐ ☐ ☐ ☐ ☐ ☐ AGREE |
| 21 | I feel comfortable communicating with the patient using the system. | ☐ | DISAGREE ☐ ☐ ☐ ☐ ☐ ☐ ☐ AGREE |
| 22 | This system is an acceptable way to provide healthcare services. | ☐ | DISAGREE ☐ ☐ ☐ ☐ ☐ ☐ ☐ AGREE |
| 23 | The discharge and insurance billing system of the platform is smooth and effective. | ☐ | DISAGREE ☐ ☐ ☐ ☐ ☐ ☐ ☐ AGREE |
| 24 | I would use HelixVM services again. | ☐ | DISAGREE ☐ ☐ ☐ ☐ ☐ ☐ ☐ AGREE |
| 25 | Overall, I am satisfied with HelixVM platform. | ☐ | DISAGREE ☐ ☐ ☐ ☐ ☐ ☐ ☐ AGREE |

**In this questionnaire, 1 - strongly disagree, 2 – disagree, 3 – somewhat disagree, 4 – neither**

**agree nor disagree, 5 – somewhat agree, 6 – agree, 7 – strongly agree**
