# Supplementary material for: Assessing the Usability and Effectiveness of an AI-Powered Telehealth Platform: Mixed Methods Study on the Perspectives of Patients and Providers
Source: JMIR Form Res. 2024 Nov 25;8:e62742. doi: 10.2196/62742 (PMC11629036; doi:10.2196/62742)
Supplement: Multimedia Appendix 2 [file formative_v8i1e62742_app2.docx]

Provider Interview Guide

| ***Question Category*** | **Q No.** | **Interview Questions and cues** |
| --- | --- | --- |
| *Introduction* | 1 | How are you liking working with HelixVM? |
| *Streamlines interaction with patients* | 2 | What do you think about asynchronous medicine as a way for improving patient access?   - data safety & privacy - quality of care - improved outcomes for patients - what did you know about asynchronous medicine before you started working with HelixVM? |
|  | 3 | How does HelixVM perform as a platform for practicing asynchronous medicine?   - Will you like something improved to enhance your efficiency of treating/interacting with patients? |
| *Streamlines record and exchange of information* | 4 | What do you think about the data record and exchange facilitated by HelixVM?   - SOAP note having expansive patient notes - subjective data (from patients) and objective (other third-party sources) - follow-up treatment notes |
|  | 5 | Is there anything missing in HelixVM SOAP notes that you would like to be added?   - Is accurate (objective) pharmacy information from a bona fide clearinghouse? - Anything about the actual patient questionnaire presented by the software to the patient during intake - Do you have any other suggestions for improvements regarding documentation? |
| *Seamless integration within EMR* | 6 | What do you think about HelixVM integration with your EMR? |
|  | 7 | Is there anything you would like to see improved in this regard?   - Would you like to have HelixVM video technology link into your EMR? - Would you like a smartphone-based app for consultations integrated in your EMR when you are away from your desktop? - Would you like to have a dropdown function to insert HelixVM SOAP note into your EMR so that you can prevent accidental publication? |
| *AI integrated triage clearing and assistance in clinical decision-making* | 8 | Are you comfortable using an AI decision support for improving patient provider interaction and medical triage?   - If you could create your own library of triage and treatment protocols, would you be more comfortable with using AI to diagnose and treat patients? |
|  | 9 | What do you think of HelixVM’s AI-integrated triage clearing capability?   - Do you think the SOAP note has all the information you need to properly diagnose and treat patients? |
|  | 10 | Are there any missing elements in the current HelixVM triage system? Would you like to any improvements?   - would you like to have accurate ICD coding and Treatment Plan suggestions? - The Helix VM Triage input should remove differential ICD codes to reduce professional liability exposure |
| *Saves Time* | 11 | On an average, how long does a patient encounter last in-person, on other telemedicine platforms and on HelixVM? |
|  | 12 | What is the most time-consuming procedure during a consultation that can be improved by over normal/current workflow?   - Documentation - Is the HelixVM triage SOAP note helpful to save time? |
|  | 13 | Do you think saving time per patient using HelixVM can contribute significantly to increasing your productivity?   - Also, how can it affect the quality of care you provide? |
| *Compensation and productivity* | 14 | How do you view the idea of working in a time-share market-place type of model to earn an extra income?   - Is compensation one of the factors? If yes, what amount of compensation would you like to have per day? - Would you like to be paid per encounter or per hour? |
|  | 15 | Would you like to make use of a platform such as HelixVM in your main practice to see more patients?   - If not, why? - If yes, how many patients do you think you can see asynchronously per hour, in your practice on a daily basis? |
