# Supplementary material for: Assessing the Usability and Effectiveness of an AI-Powered Telehealth Platform: Mixed Methods Study on the Perspectives of Patients and Providers
Source: JMIR Form Res. 2024 Nov 25;8:e62742. doi: 10.2196/62742 (PMC11629036; doi:10.2196/62742)
Supplement: Multimedia Appendix 3 [file formative_v8i1e62742_app3.docx]

|  | **Cronbach Alpha original TUQ** | **Cronbach Alpha modified TUQ** |
| --- | --- | --- |
| Usefulness | 0.85 | 0.792 |
| Ease of use | 0.93 | 0.927 |
| Effectiveness | 0.87 | 0.872 |
| Reliability | 0.81 | 0.708 |
| Satisfaction | 0.92 | 0.848 |

| **#** | **Statements** | **Sub-scales** |
| --- | --- | --- |
| 1 | HelixVM improves my access to healthcare services. | Usefulness |
| 2 | The platform saved me time traveling to a doctor, hospital or a specialist clinic. |  |
| 3 | The platform provides for my healthcare need. |  |
| 4 | I find the Fast-track Rx (ability to receive prescription without virtual visit) service useful. |  |
| 5 | It was simple to use this system. | Ease of Use |
| 6 | It was easy to learn to use the system. |  |
| 7 | The way I interact with this system is pleasant. |  |
| 8 | I like using the system. |  |
| 9 | The system is simple and easy to understand. |  |
| 10 | I believe I could receive the care I needed quickly using this system. | Effectiveness |
| 11 | This system can do everything I would want it to be able to do. |  |
| 12 | I can easily talk to the clinician and/or receive treatment using this system. |  |
| 13 | I can hear the clinician clearly using the telehealth system. |  |
| 14 | I felt I was able to express myself effectively. |  |
| 15 | Using the system, I can see the clinician as well as if we met in person. |  |
| 16 | Whenever I made a mistake using the system, I could recover easily and quickly. | Reliability |
| 17 | The system gave error messages that clearly told me how to fix problems. |  |
| 18 | I feel comfortable communicating with the clinician using the system. | Satisfaction |
| 19 | This app/platform is an acceptable way to receive healthcare services. |  |
| 20 | I would use the HelixVM services again. |  |
| 21 | Overall, I am satisfied with HelixVM. |  |
