# Supplementary material for: Assessing the Usability and Effectiveness of an AI-Powered Telehealth Platform: Mixed Methods Study on the Perspectives of Patients and Providers
Source: JMIR Form Res. 2024 Nov 25;8:e62742. doi: 10.2196/62742 (PMC11629036; doi:10.2196/62742)
Supplement: Multimedia Appendix 4 [file formative_v8i1e62742_app4.docx]

| **S.No.** | **Variable** | **Category Name (code)** | **Observed Frequencies** | **P-value Overall** |
| --- | --- | --- | --- | --- |
| 1 | HelixVM improves my access to healthcare services | Strongly Disagree (1) | 4 | <0.001 |
|  |  | Neither Agree nor Disagree (4) | 5 |  |
|  |  | Somewhat Agree (5) | 15 |  |
|  |  | Agree (6) | 40 |  |
|  |  | Strong Agree (7) | 38 |  |
| 2 | The platform saved me time travelling to a doctor, hospital or a specialist clinic | Somewhat Disagree (3) | 2 | <0.001 |
|  |  | Somewhat Agree (5) | 10 |  |
|  |  | Agree (6) | 26 |  |
|  |  | Strongly Agree (7) | 64 |  |
| 3 | The platform provides for my healthcare need | Strongly Disagree (1) | 2 | <0.001 |
|  |  | Disagree (2) | 2 |  |
|  |  | Somewhat Disagree (3) | 3 |  |
|  |  | Neither Agree nor Disagree (4) | 4 |  |
|  |  | Somewhat Agree (5) | 18 |  |
|  |  | Agree (6) | 32 |  |
|  |  | Strong Agree (7) | 41 |  |
| 4 | It was simple to use this platform | Disagree (2) | 2 | <0.001 |
|  |  | Somewhat Disagree (3) | 5 |  |
|  |  | Neither Agree nor Disagree (4) | 3 |  |
|  |  | Somewhat Agree (5) | 19 |  |
|  |  | Agree (6) | 30 |  |
|  |  | Strong Agree (7) | 43 |  |
| 5 | It was easy to learn to use the system | Disagree (2) | 2 | <0.001 |
|  |  | Somewhat Disagree (3) | 3 |  |
|  |  | Neither Agree nor Disagree (4) | 4 |  |
|  |  | Somewhat Agree (5) | 16 |  |
|  |  | Agree (6) | 35 |  |
|  |  | Strongly Agree (7) | 42 |  |
| 6 | I believe I could receive the care I needed quickly using this system | Strongly Disagree (1) | 2 | <0.001 |
|  |  | Disagree (2) | 1 |  |
|  |  | Somewhat Disagree (3) | 18 |  |
|  |  | Neither Agree nor Disagree (4) | 5 |  |
|  |  | Agree (6) | 37 |  |
|  |  | Strongly Agree (7) | 39 |  |
| 7 | The way I interact with this system is pleasant | Strongly Disagree (1) | 1 | <0.001 |
|  |  | Disagree (2) | 2 |  |
|  |  | Somewhat Disagree (3) | 4 |  |
|  |  | Neither Agree nor Disagree (4) | 7 |  |
|  |  | Somewhat Agree (5) | 16 |  |
|  |  | Agree (6) | 37 |  |
|  |  | Strongly Agree (7) | 35 |  |
| 8 | I like using this system | Strongly Disagree (1) | 2 | <0.001 |
|  |  | Disagree (2) | 3 |  |
|  |  | Somewhat Disagree (3) | 4 |  |
|  |  | Neither Agree nor Disagree (4) | 6 |  |
|  |  | Somewhat Agree (5) | 15 |  |
|  |  | Agree (6) | 39 |  |
|  |  | Strongly Agree (7) | 33 |  |
| 9 | The system is simple and easy to understand | Disagree (2) | 2 | <0.001 |
|  |  | Somewhat Disagree (3) | 2 |  |
|  |  | Neither Agree nor Disagree (4) | 6 |  |
|  |  | Somewhat Agree (5) | 22 |  |
|  |  | Agree (6) | 34 |  |
|  |  | Strongly Agree (7) | 36 |  |
| 10 | This system can do everything I would want it to be able to | Strongly Disagree (1) | 2 | <0.001 |
|  |  | Disagree (2) | 4 |  |
|  |  | Somewhat Disagree (3) | 13 |  |
|  |  | Neither Agree nor Disagree (4) | 8 |  |
|  |  | Somewhat Agree (5) | 17 |  |
|  |  | Agree (6) | 27 |  |
|  |  | Strongly Agree (7) | 31 |  |
| 11 | I can easily talk to the clinician and/or receive treatment using this system | Strongly Disagree (1) | 3 | <0.001 |
|  |  | Disagree (2) | 9 |  |
|  |  | Somewhat Disagree (3) | 3 |  |
|  |  | Neither Agree nor Disagree (4) | 4 |  |
|  |  | Somewhat Agree (5) | 11 |  |
|  |  | Agree (6) | 33 |  |
|  |  | Strongly Agree (7) | 39 |  |
| 12 | I can hear the clinician clearly using the telehealth system | Strongly Disagree (1) | 1 | <0.001 |
|  |  | Disagree (2) | 2 |  |
|  |  | Somewhat Disagree (3) | 1 |  |
|  |  | Neither Agree nor Disagree (4) | 17 |  |
|  |  | Somewhat Agree (5) | 4 |  |
|  |  | Agree (6) | 34 |  |
|  |  | Strongly Agree (7) | 33 |  |
| 13 | I was able to express myself effectively | Strongly Disagree (1) | 1 | <0.001 |
|  |  | Disagree (2) | 2 |  |
|  |  | Somewhat Disagree (3) | 1 |  |
|  |  | Neither Agree nor Disagree (4) | 9 |  |
|  |  | Somewhat Agree (5) | 10 |  |
|  |  | Agree (6) | 38 |  |
|  |  | Strongly Agree (7) | 41 |  |
| 14 | Using the system, I can see the clinician as well as if we met in person | Strongly Disagree (1) | 2 | <0.001 |
|  |  | Disagree (2) | 4 |  |
|  |  | Somewhat Disagree (3) | 2 |  |
|  |  | Neither Agree nor Disagree (4) | 16 |  |
|  |  | Somewhat Agree (5) | 5 |  |
|  |  | Agree (6) | 35 |  |
|  |  | Strongly Agree (7) | 38 |  |
| 15 | The system gave error messages that clearly told me how to fix problems | Strongly Disagree (1) | 2 | <0.001 |
|  |  | Disagree (2) | 4 |  |
|  |  | Somewhat Disagree (3) | 2 |  |
|  |  | Neither Agree nor Disagree (4) | 16 |  |
|  |  | Somewhat Agree (5) | 5 |  |
|  |  | Agree (6) | 35 |  |
|  |  | Strongly Agree (7) | 38 |  |
| 16 | Whenever I made a mistake using the system, I could recover easily and quickly | Strongly Disagree (1) | 2 | <0.001 |
|  |  | Disagree (2) | 1 |  |
|  |  | Somewhat Disagree (3) | 2 |  |
|  |  | Neither Agree nor Disagree (4) | 19 |  |
|  |  | Somewhat Agree (5) | 16 |  |
|  |  | Agree (6) | 31 |  |
|  |  | Strongly Agree (7) | 31 |  |
| 17 | I find the fast-track Rx (ability to receive prescription without virtual visit) service useful | Strongly Disagree (1) | 4 | <0.001 |
|  |  | Disagree (2) | 1 |  |
|  |  | Somewhat Disagree (3) | 1 |  |
|  |  | Neither Agree nor Disagree (4) | 5 |  |
|  |  | Somewhat Agree (5) | 5 |  |
|  |  | Agree (6) | 19 |  |
|  |  | Strongly Agree (7) | 67 |  |
| 18 | I feel comfortable communicating with the clinician using the system | Strongly Disagree (1) | 1 | <0.001 |
|  |  | Somewhat Disagree (3) | 2 |  |
|  |  | Neither Agree nor Disagree (4) | 8 |  |
|  |  | Somewhat Agree (5) | 8 |  |
|  |  | Agree (6) | 36 |  |
|  |  | Strongly Agree (7) | 47 |  |
| 19 | This app/platform is an acceptable way to receive healthcare services. | Strongly Disagree (1) | 1 | <0.001 |
|  |  | Somewhat Disagree (3) | 4 |  |
|  |  | Neither Agree nor Disagree (4) | 4 |  |
|  |  | Somewhat Agree (5) | 7 |  |
|  |  | Agree (6) | 32 |  |
|  |  | Strongly Agree (7) | 54 |  |
| 20 | I would use the HelixVM services again | Strongly Disagree (1) | 3 | <0.001 |
|  |  | Disagree (2) | 5 |  |
|  |  | Neither Agree nor Disagree (4) | 3 |  |
|  |  | Somewhat Agree (5) | 9 |  |
|  |  | Agree (6) | 23 |  |
|  |  | Strongly Agree (7) | 59 |  |
| 21 | Overall, I am satisfied with HelixVM | Strongly Disagree (1) | 2 | <0.001 |
|  |  | Disagree (2) | 3 |  |
|  |  | Somewhat Disagree (3) | 2 |  |
|  |  | Neither Agree nor Disagree (4) | 7 |  |
|  |  | Somewhat Agree (5) | 10 |  |
|  |  | Agree (6) | 29 |  |
|  |  | Strongly Agree (7) | 49 |  |
| 22 | On a scale of 1 to 10, how would you rate yourself on tech savviness | 0 | 8 | <0.001 |
|  |  | 1 | 9 |  |
|  |  | 2 | 12 |  |
|  |  | 3 | 7 |  |
|  |  | 4 | 1 |  |
|  |  | 5 | 4 |  |
|  |  | 6 | 3 |  |
|  |  | 7 | 9 |  |
|  |  | 8 | 21 |  |
|  |  | 9 | 7 |  |
|  |  | 10 | 21 |  |
