# Supplementary figures and images for: Assessing the Usability and Effectiveness of an AI-Powered Telehealth Platform: Mixed Methods Study on the Perspectives of Patients and Providers
Source: JMIR Form Res. 2024 Nov 25;8:e62742. doi: 10.2196/62742 (PMC11629036; doi:10.2196/62742)

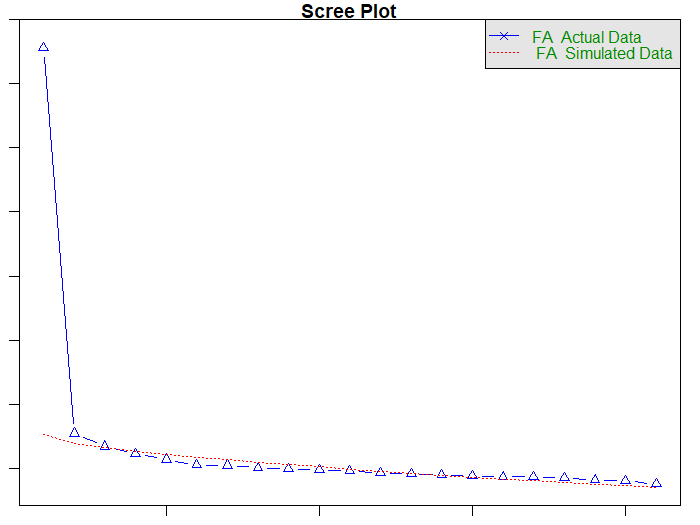

Supplement: Multimedia Appendix 5 [file formative_v8i1e62742_app5.docx]

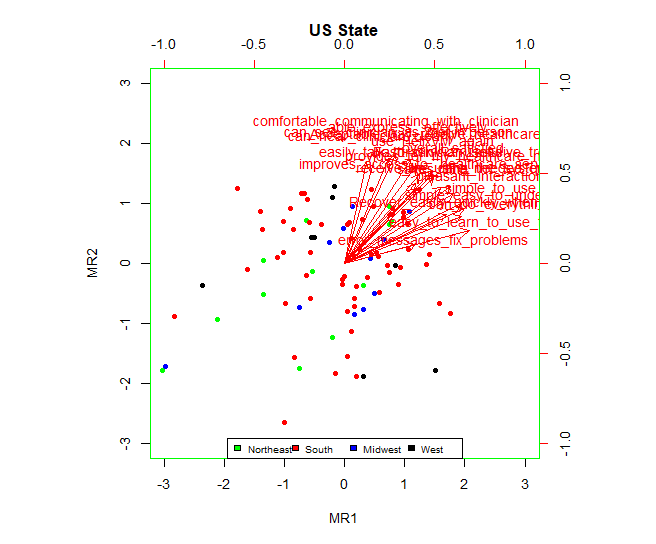

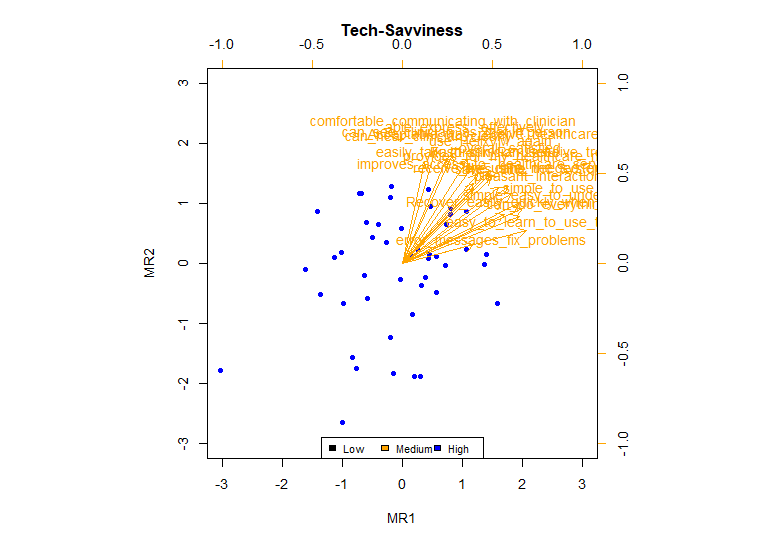

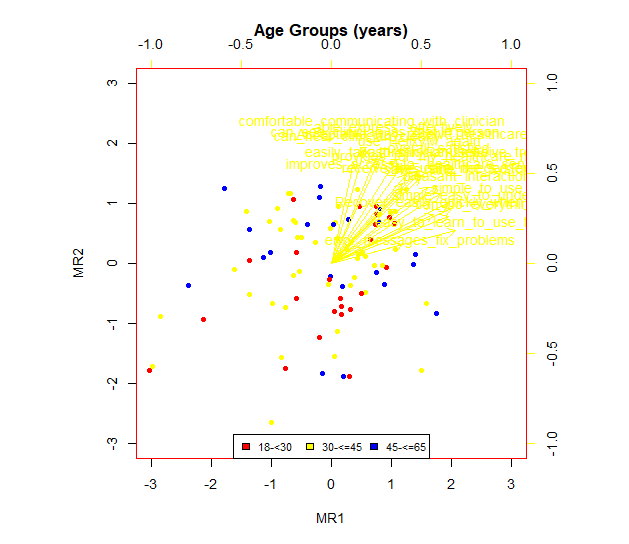

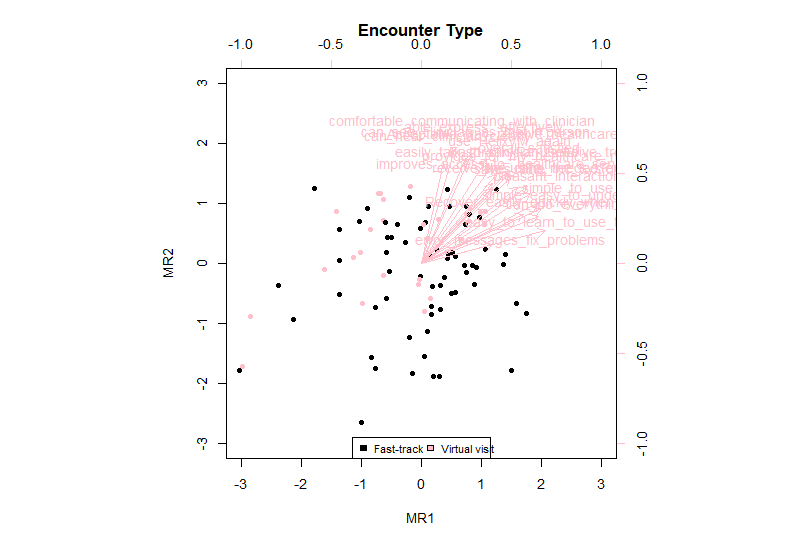

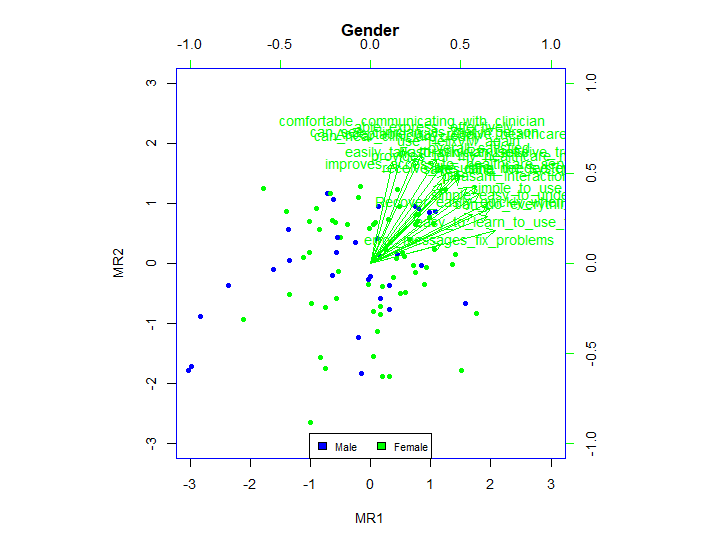

Supplement: Multimedia Appendix 6 [file formative_v8i1e62742_app6.docx]
